# Supplementary material for: The effect of using group-averaged or individualized brain parcellations when investigating connectome dysfunction in psychosis
Source: Netw Neurosci. 2023 Dec 22;7(4):1228–47. doi: 10.1162/netn_a_00329 (PMC10631788; doi:10.1162/netn_a_00329)
Supplement: Supplementary file 1 [file netn-7-4-1228-s001.pdf]

Supplementary Materials

Spatial and functional properties of group-based vs individualized parcellation

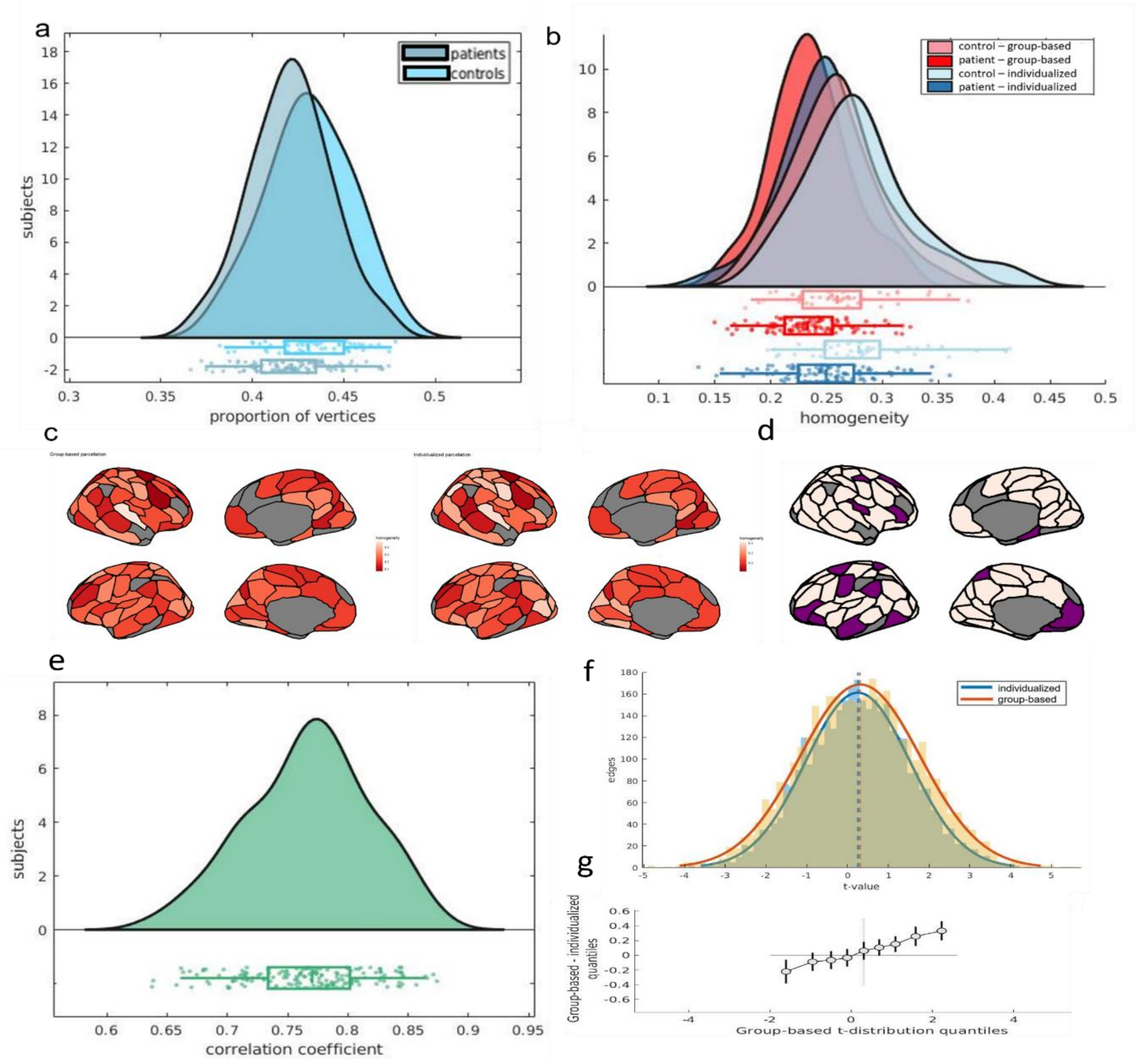

Figure 1 - Spatial and functional properties of group-based vs individualized parcellation, based on s100 GSR. a The proportion of vertices changed for controls $\left(M(SD) = 0.431(0.023)\right)$ and for patients $\left(M(SD) = 0.421(0.022)\right)$. There were slightly more vertices relabelled in controls than in patients $t(165) = 2.448, p = 0.0077\ 95\%CI = [0.003, 0.017]$. **b** The distribution of homogeneity scores per subject. Mean homogeneity for the group

parcellation in controls was 0.261 ($SD = 0.04$), and 0.281 ($SD = 0.05$) for the individualized parcellation. In patients, the mean homogeneity for the group parcellation was 0.235 ($SD = 0.04$) and 0.250 ($SD = 0.04$) for the individualized parcellation. A two-way mixed ANOVA revealed that mean homogeneity was higher for the individualized parcellation ($F(148) = 234.91, p < 0.0001$) and higher in controls compared to patients ($F(148) = 15.72, p = 0.0001$), with an interaction between parcellation type and diagnosis ($F(148) = 4.68, p = 0.032$). Panel **c** shows homogeneity scores for every parcel for group-based and individualized parcellation. Light colored parcels in **d** represent parcels showing significant difference in homogeneity scores, between parcellation approaches, for $p_{FDR} < 0.05$. Homogeneity is displayed in inflated surfaces with the group-based parcellation. **e** The distribution of the Pearson's coefficient of correlation comparing FC matrices derived from group-based and individualized parcellation. Matrices were positively correlated and ranged between 0.637 and 0.874 (median = 0.770). **f** Distributions of t-values quantifying FC differences between patients and controls at each edge and for individualized parcellation $\left(M(SD) = 0.253(1.28)\right)$ and for group-based parcellation $\left(M(SD) = 0.310(1.48)\right)$. The difference between the individualized and group-based parcellations were statistically significant, according to a Wilcoxon Sign Rank Test ($Z = 3.471, p < 0.0001$). **g** Shift function for the two t-distributions. Each circle represents the difference between each decile of both distributions, as a function of the deciles in group-based distribution and the bars represent the 95% boot-strap confidence interval associated with the difference.

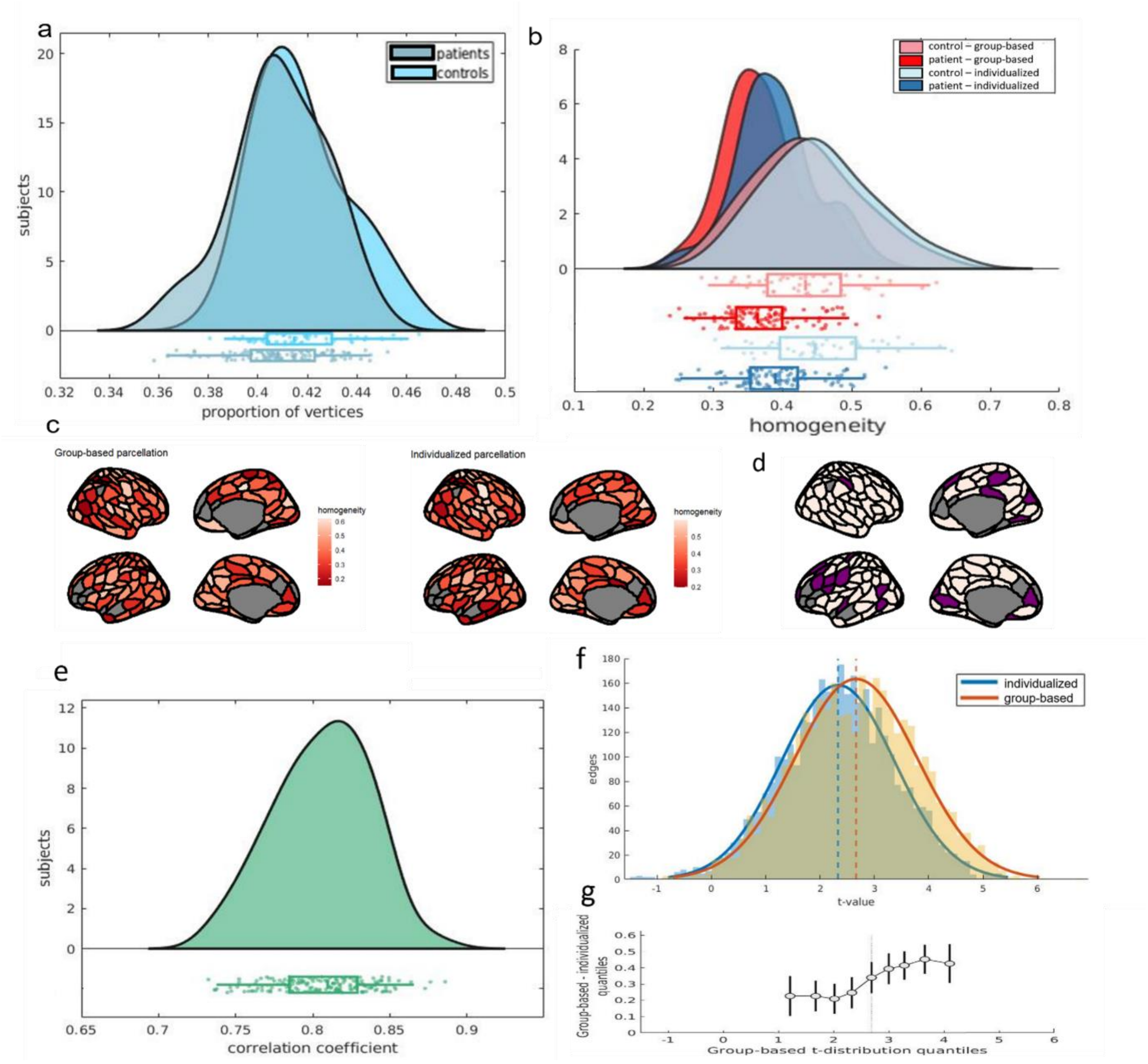

Figure 2 - Spatial and functional properties of group-based vs individualized parcellation, based on s200. a The proportion of vertices changed for controls $\left(M(SD) = 0.418(0.019)\right)$ and for patients $\left(M(SD) = 0.409(0.020)\right)$. There were slightly more vertices relabelled in controls than in patients $t(163) = 2.448, p = 0.0078$ $95\%CI = [0.002, 0.015]$. **b** The distribution of homogeneity scores per subject. Mean homogeneity for the group parcellation in controls was 0.434 $(SD = 0.08)$, and 0.454 $(SD = 0.08)$ for the individualized parcellation. In patients, the mean homogeneity for the group parcellation was 0.371 $(SD = 0.06)$ and 0.392 $(SD = 0.06)$ for the individualized parcellation. A two-way mixed ANOVA revealed that mean homogeneity was higher for the individualized parcellation $(F(148) =$

$901.60, p < 0.0001$) and higher in controls compared to patients ($F(148) = 29.33, p < 0.0001$), with no interaction between parcellation type and diagnosis ($F(148) = 0.708, p = 0.402$). Panel **c** shows homogeneity scores for every parcel for group-based and individualized parcellation. Light colored parcels in **d** represent parcels showing significant difference in homogeneity scores, between parcellation approaches, for $p_{FDR} < 0.05$. Homogeneity is displayed in inflated surfaces with the group-based parcellation. **e** The distribution of the Pearson's coefficient of correlation comparing FC matrices derived from group-based and individualized parcellation. Matrices were positively correlated and ranged between 0.732 and 0.886 (median = 0.810). **f** Distributions of t-values quantifying FC differences between patients and controls at each edge and for individualized parcellation $\left(M(SD) = 2.330(1.04)\right)$ and for group-based parcellation $\left(M(SD) = 2.663(1.13)\right)$. The difference between the individualized and group-based parcellations were statistically significant, according to a Wilcoxon Sign Rank Test ($Z = 24.053, p < 0.0001$). **g** Shift function for the two t-distributions. Each circle represents the difference between each decile of both distributions, as a function of the deciles in group-based distribution and the bars represent the 95% boot-strap confidence interval associated with the difference.

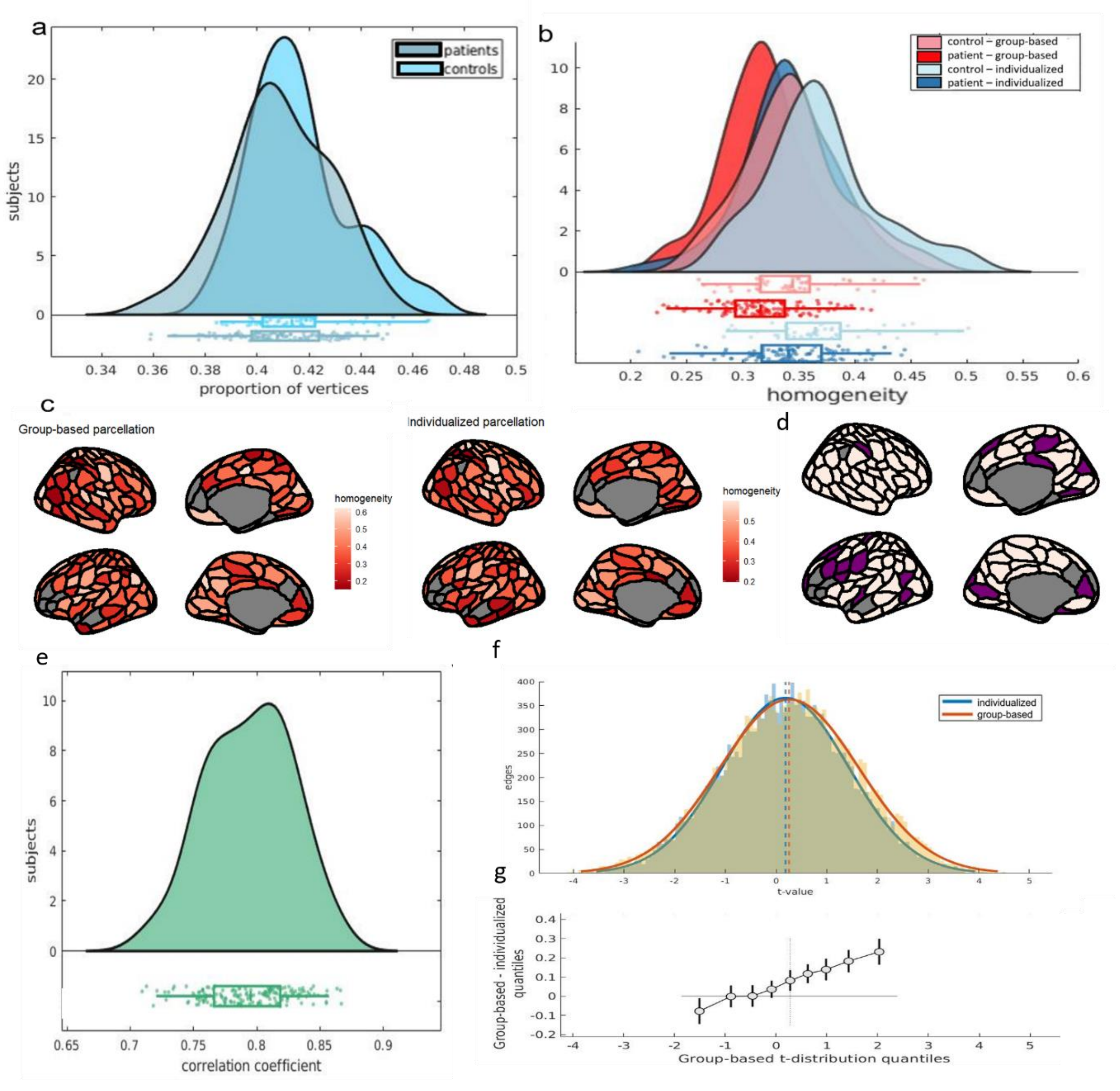

Figure 3 - Spatial and functional properties of group-based vs individualized parcellation, based on s200 GSR. a The proportion of vertices changed for controls $\left(M(SD) = 0.416(0.019)\right)$ and for patients $\left(M(SD) = 0.409(0.019)\right)$. There were slightly more vertices relabelled in controls than in patients $t(163) = 2.479, p = 0.0071\ 95\%CI = [0.001, 0.014]$. **b** The distribution of homogeneity scores per subject. Mean homogeneity for the group parcellation in controls was 0.346 ($SD = 0.05$), and 0.370 ($SD = 0.05$) for the individualized parcellation. In patients, the mean homogeneity for the group parcellation was 0.318 ($SD = 0.04$) and 0.341 ($SD = 0.04$) for the individualized parcellation. A two-way mixed ANOVA revealed that mean homogeneity was higher for the individualized parcellation ($F(148) =$

$1040.06, p < 0.0001$) and higher in controls compared to patients ($F(148) = 14.35, p = 0.0002$), with no interaction between parcellation type and diagnosis ($F(148) = 0.246, p = 0.621$). Panel **c** shows homogeneity scores for every parcel for group-based and individualized parcellation. Light colored parcels in **d** represent parcels showing significant difference in homogeneity scores, between parcellation approaches, for $p_{FDR} < 0.05$. Homogeneity is displayed in inflated surfaces with the group-based parcellation. **e** The distribution of the Pearson's coefficient of correlation comparing FC matrices derived from group-based and individualized parcellation. Matrices were positively correlated and ranged between 0.710 and 0.867 (Median = 0.795). **f** Distributions of t-values quantifying FC differences between patients and controls at each edge and for individualized parcellation $\left(M(SD) = 0.184(1.25)\right)$ and for group-based parcellation $\left(M(SD) = 0.261(1.37)\right)$. The difference between the individualized and group-based parcellations were statistically significant, according to a Wilcoxon Sign Rank Test ($Z = 10.581, p < 0.0001$). **g** Shift function for the two t-distributions. Each circle represents the difference between each decile of both distributions, as a function of the deciles in group-based distribution and the bars represent the 95% boot-strap confidence interval associated with the difference.

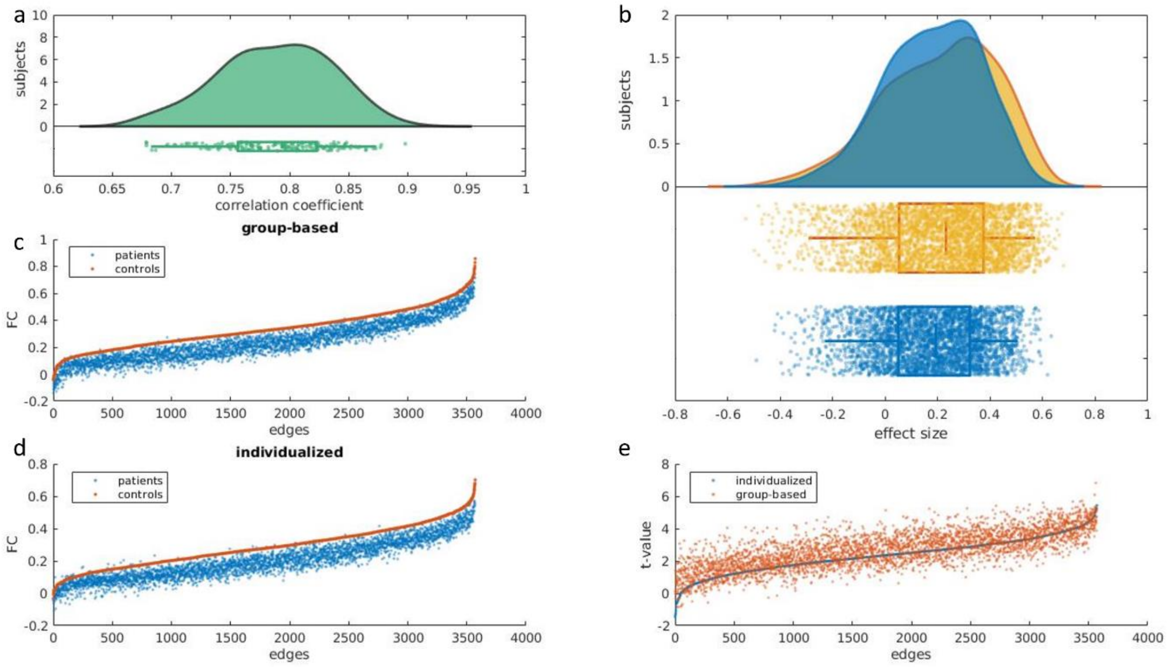

Figure 4 – Correlation between FC matrices derived from different parcellation approaches, based on the s100 atlas. Panel **a** shows the distribution and boxplot of correlations between FC matrices derived using group-based and individualized parcellations for each individual. Panel **b** shows the effect size (Cohen's *d*) of the FC differences between patients and controls at every edge, as observed using both parcellation approaches. Panels **c** and **d** show the average FC for every edge for patients and controls, ordered by controls FC values. Panel **e** shows the t-values associated with patients and controls FC differences, ordered by the individualized parcellation t-values.

Thresholded group differences in FC according to parcellation method

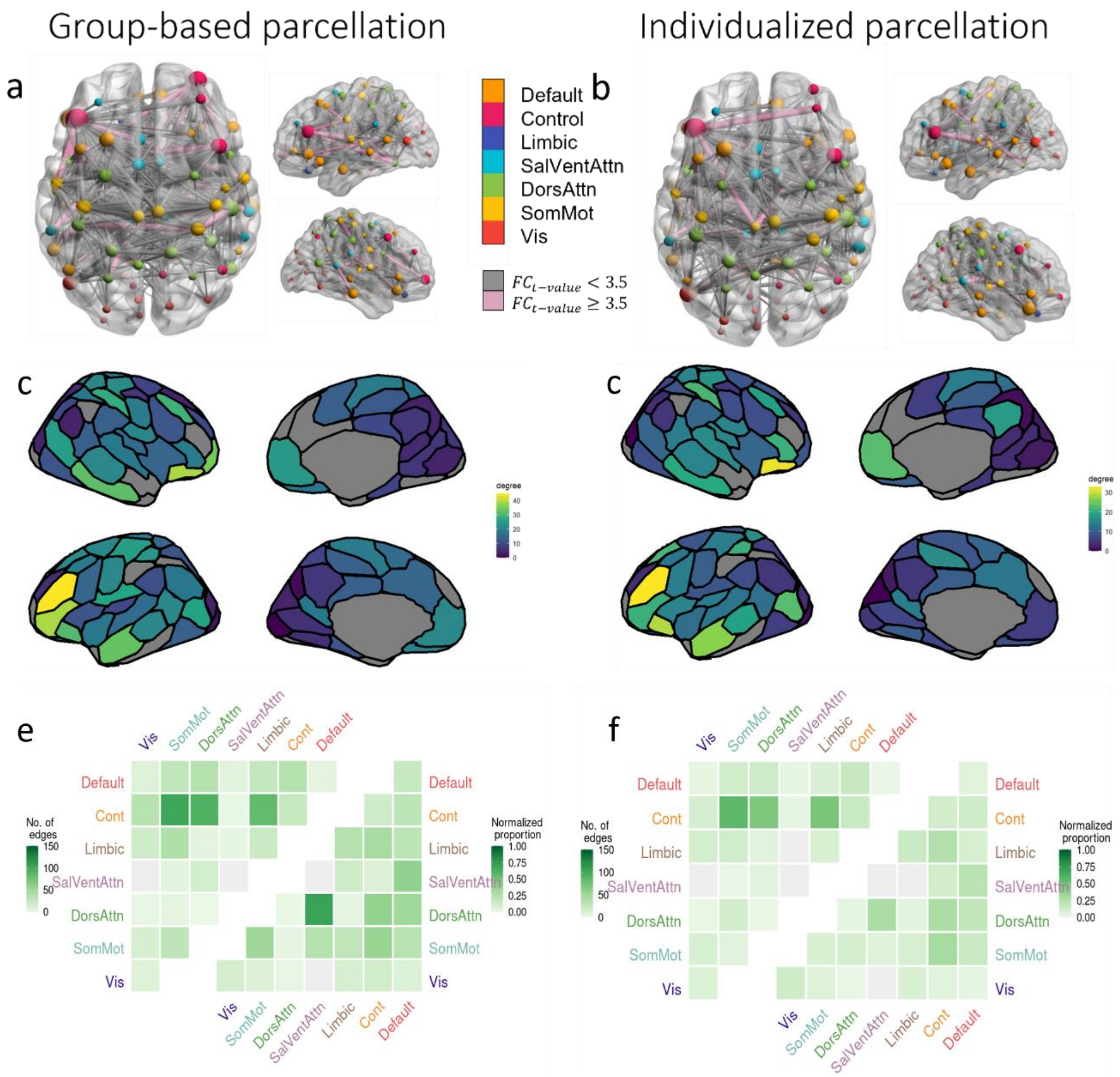

Figure 5 – Edge-level regional and network-level case-control FC differences according to parcellation type, based on s100 GSR. The NBS identified a single connected component as showing significant FC differences between groups using both the (**a**) group-based ($p = 0$) and (**b**) individualized parcellations ($p = 0.0002$). The group-based component (**a** and **c**) comprises 676 edges and the individualized component (**b** and **d**) comprises 484 edges. Panels

a and **b** show the specific edges comprising the NBS components obtained with the group-based and individualized parcellations, respectively, with nodes colored according to network affiliation and sized by degree. Edges are sized by strength of dysconnectivity. Edges associated with a t-value < 3.5 are represented by grey lines and those associated with a t-value ≥ 3.5 are represented in pink. The images were created using the software BrainNet Viewer (Xia et al., 2013). Panels **a**, **c** and **e** are based on group parcellation. Panels **c** and **d** show the degree of each region in the NBS component for the group and individualized parcellations, respectively. Edges are represented by grey lines. The upper triangle of each matrix in panels **e** and **f** shows the total number of NBS component edges (raw counts) falling within and between seven canonical networks. The lower triangles show the same data normalized for network size (normalized counts). Vis – visual network; SomMot – somatomotor network; DorsAttn – dorsal attention network; SalVentAttn – salience/ventral attention network; Cont – control network; Default – Default Mode Network.

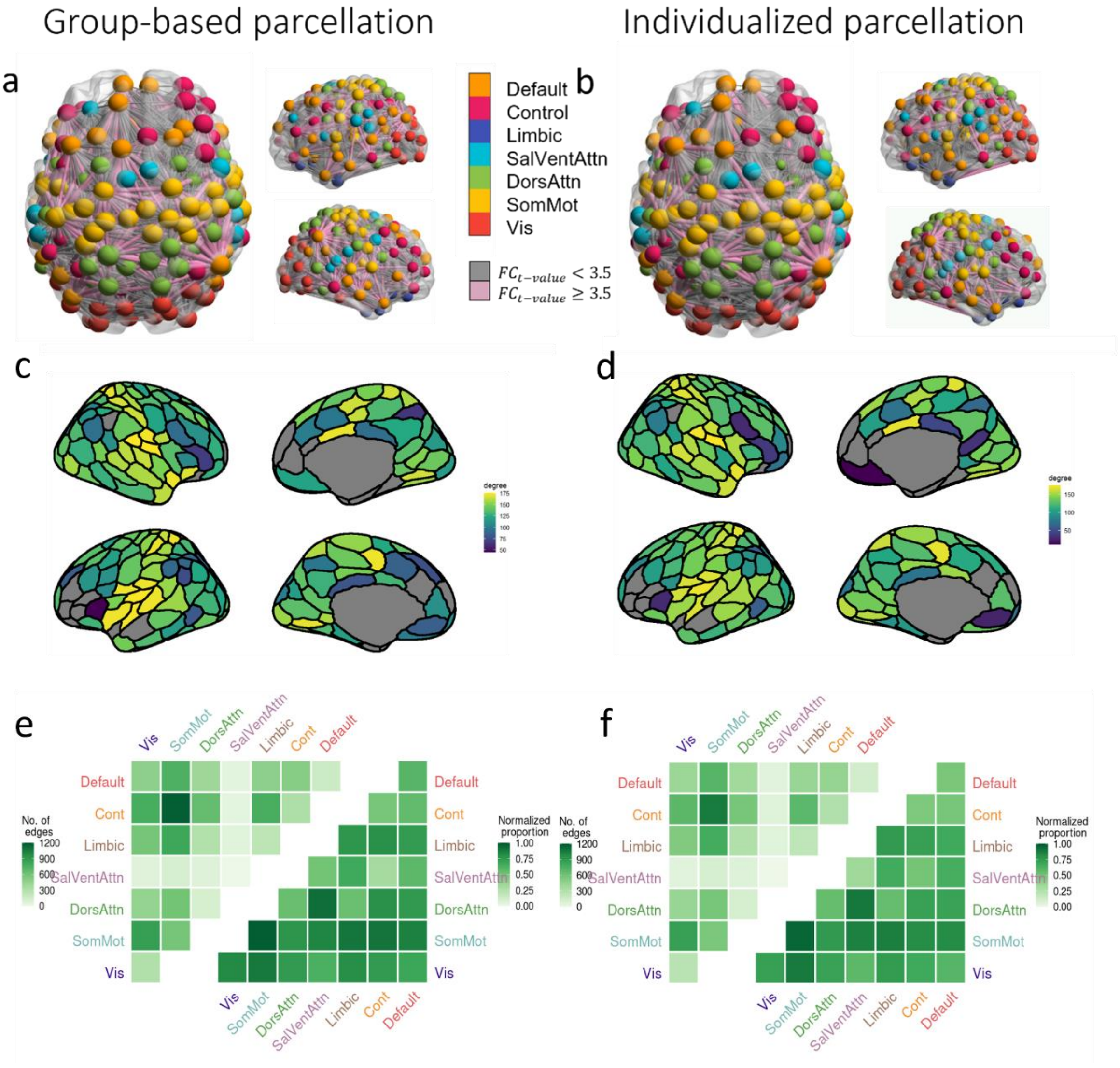

Figure 6 – Edge-level regional and network-level case-control FC differences according to parcellation type, based on s200. The NBS identified a single connected component as showing significant FC differences between groups using both the (**a**) group-based $(p = 0;\ \text{i.e.,}$ no null value exceeded the observed estimate$)$ and (**b**) individualized parcellations ($p = 0$). The group-based component (**a** and **c**) comprises 11,927 edges and the individualized component (**b** and **d**) comprises 11,149 edges. Panels **a** and **b** show the specific edges comprising the NBS components obtained with the group-based and individualized parcellations, respectively, with nodes colored according to network affiliation and sized by

degree. Edges are sized by strength of dysconnectivity. Edges associated with a t-value < 3.5 are represented by grey lines and those associated with a t-value ≥ 3.5 are represented in pink. The images were created using the software BrainNet Viewer (Xia et al., 2013). Panels **a**, **c** and **e** are based on group parcellation. Panels **c** and **d** show the degree of each region in the NBS component for the group and individualized parcellations, respectively. Edges are represented by grey lines. The upper triangle of each matrix in panels **e** and **f** shows the total number of NBS component edges (raw counts) falling within and between seven canonical networks. The lower triangles show the same data normalized for network size (normalized counts). Vis – visual network; SomMot – somatomotor network; DorsAttn – dorsal attention network; SalVentAttn – salience/ventral attention network; Cont – control network; Default – Default Mode Network.

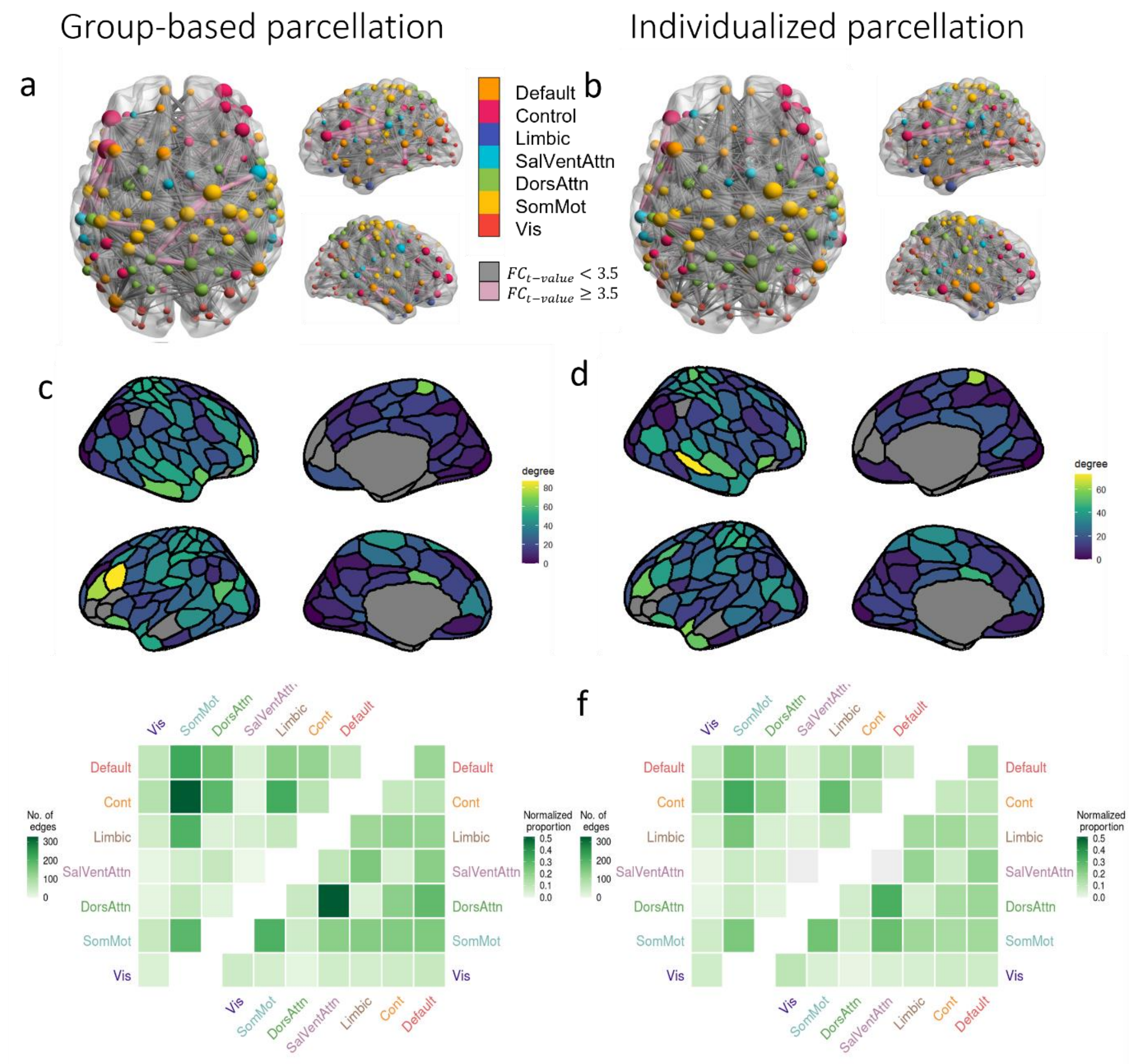

Figure 7 – Edge-level regional and network-level case-control FC differences according to parcellation type, based on s200 GSR. The NBS identified a single connected component as showing significant FC differences between groups using both the (**a**) group-based ($p = 0.001$) and (**b**) individualized parcellations ($p = 0.0014$). The group-based component (**a** and **c**) comprises 2,481 edges and the individualized component (**b** and **d**) comprises 1,882 edges. Panels **a** and **b** show the specific edges comprising the NBS components obtained with the group-based and individualized parcellations, respectively, with nodes colored according to network affiliation and sized by degree. Edges are sized by strength of dysconnectivity. Edges

associated with a t-value < 3.5 are represented by grey lines and those associated with a t-value ≥ 3.5 are represented in pink. The images were created using the software BrainNet Viewer (Xia et al., 2013). Panels **a**, **c** and **e** are based on group parcellation. Panels **c** and **d** show the degree of each region in the NBS component for the group and individualized parcellations, respectively. Edges are represented by grey lines. The upper triangle of each matrix in panels **e** and **f** shows the total number of NBS component edges (raw counts) falling within and between seven canonical networks. The lower triangles show the same data normalized for network size (normalized counts). Vis – visual network; SomMot – somatomotor network; DorsAttn – dorsal attention network; SalVentAttn – salience/ventral attention network; Cont – control network; Default – Default Mode Network.

The effects of variation in parcel size

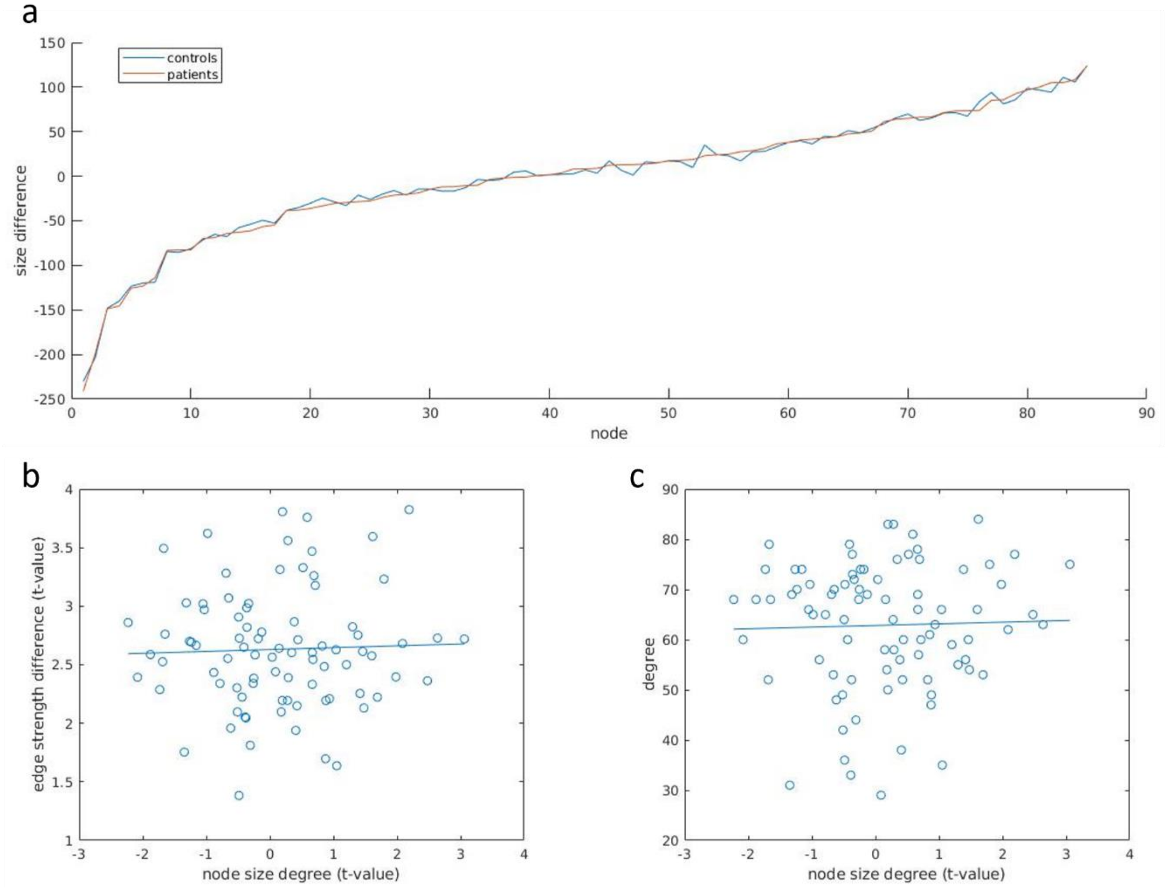

Figure 8 – Changes in parcels size and its correlation to node degree and edge dysconnectivity, based on s100. a The average difference in size of every region between individualized and group-based parcellation for patients and controls. Size is measured in terms of vertices and the change reported is the average size difference for controls and patients. Positive numbers correspond to the node being larger in individualized parcellation. There was no difference in parcel size changes between groups ($p = 0.889$) Panel **b** shows the node size difference and average edge dysconnectivity for every region (blue dots) and the correlation between both (blue line) for individualized parcellation. Panel **c** shows the node size difference and node degree for every node (blue dots) and the correlation between both (blue line) for individualized parcellation. Correlation is given by the Spearman's coefficient.

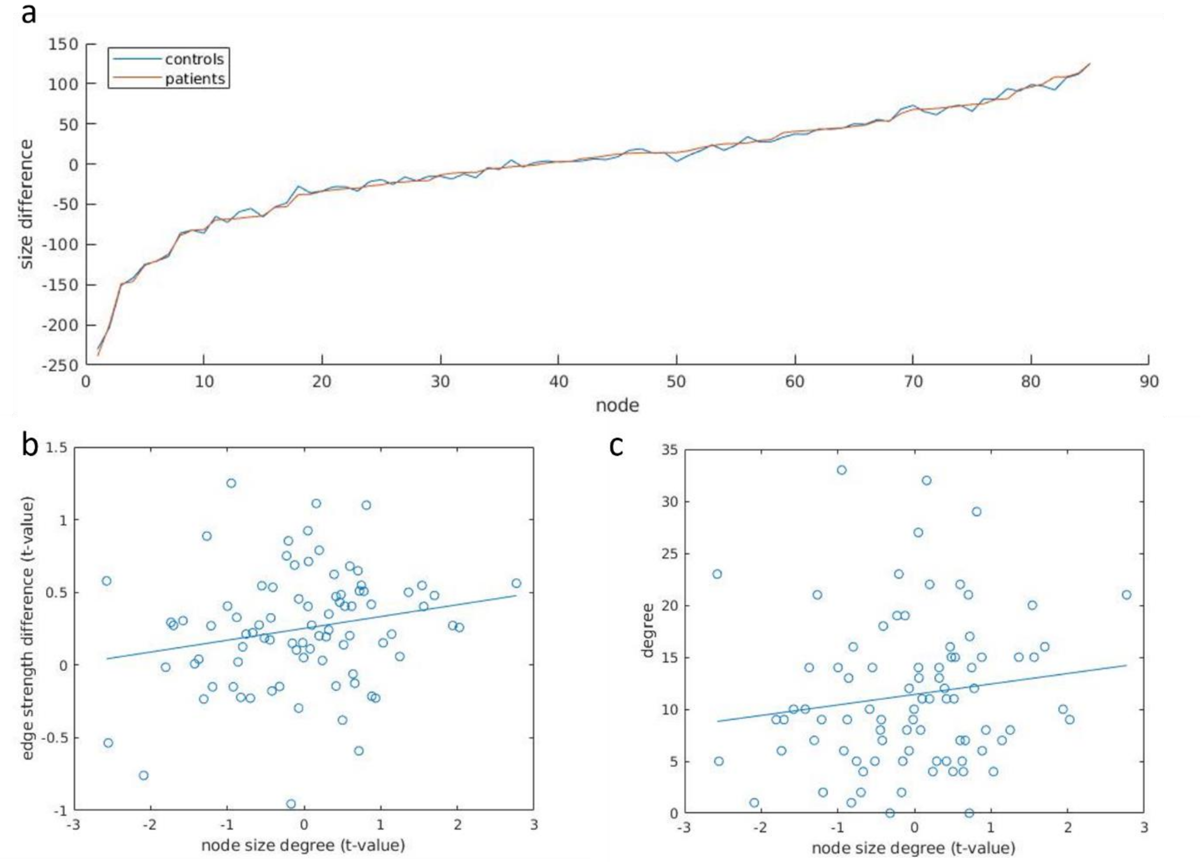

Figure 9 – Changes in parcels size and its correlation to node degree and edge dysconnectivity, based on s100 GSR. a The average difference in size of every region between individualized and group-based parcellation for patients and controls. Size is measured in terms of vertices and the change reported is the average size difference for controls and patients. Positive numbers correspond to the node being larger in individualized parcellation. There was no difference in parcel size changes between groups ($p = 0.990$). Panel **b** shows the node size difference and average edge dysconnectivity for every region (blue dots) and the correlation ($r = 0.202, p = 0.064$) between both (blue line) for individualized parcellation. Panel **c** shows the node size difference and node degree for every node (blue dots) and the correlation ($r = 0.164, p = 0.133$) between both (blue line) for individualized parcellation. Correlation is given by the Spearman's coefficient.

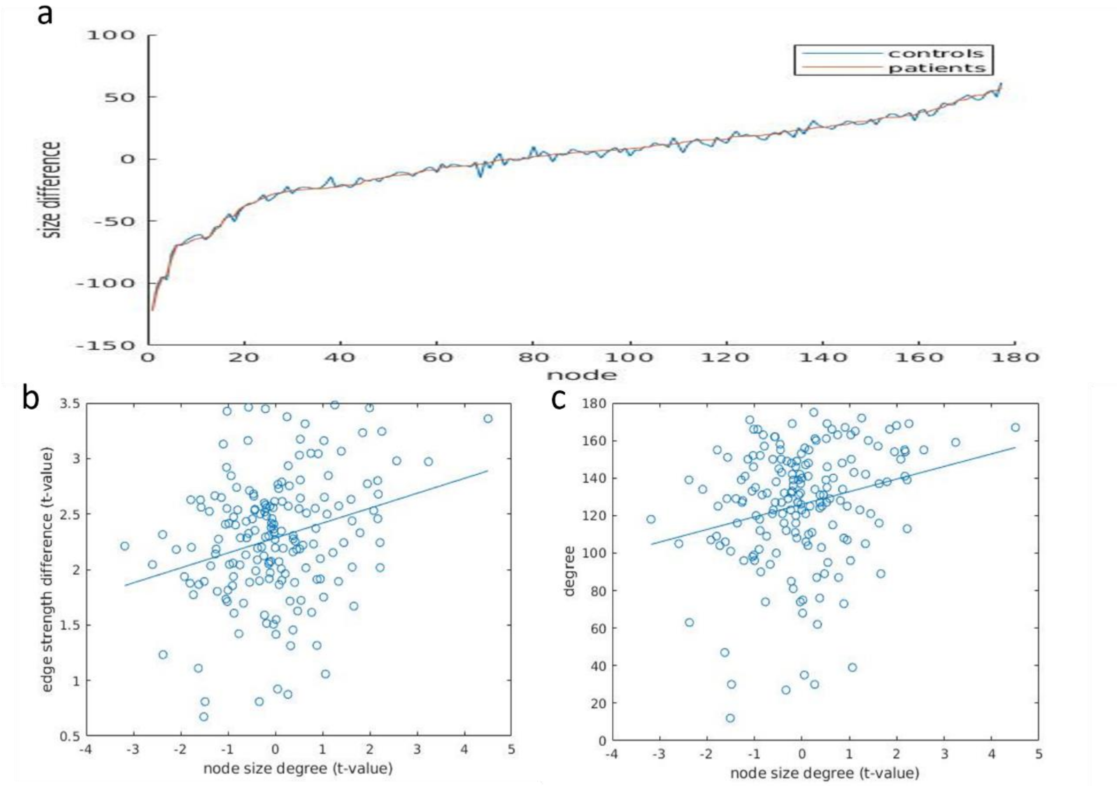

Figure 10 - Changes in parcels size and its correlation to node degree and edge dysconnectivity, based on s200. a The average difference in size of every region between individualized and group-based parcellation for patients and controls. Size is measured in terms of vertices and the change reported is the average size difference for controls and patients. Positive numbers correspond to the node being larger in individualized parcellation. There was no difference in parcel size changes between groups, according to permutation testing $(p = 0.550)$. The second region of the somatomotor network of the left hemisphere showed significant difference in change in size $(p_{FDR} = 0.035)$, with controls having a greater size difference between parcellations. Panel **b** shows the node size difference and average edge dysconnectivity for every region (blue dots) and the correlation $(r = 0.213, p = 0.004)$ between both (blue line) for individualized parcellation. Panel **c** shows the node size difference and node degree for every node (blue dots) and the correlation $(r = 0.215, p =$

0.004) between both (blue line) for individualized parcellation. Correlation is given by the Spearman's coefficient.

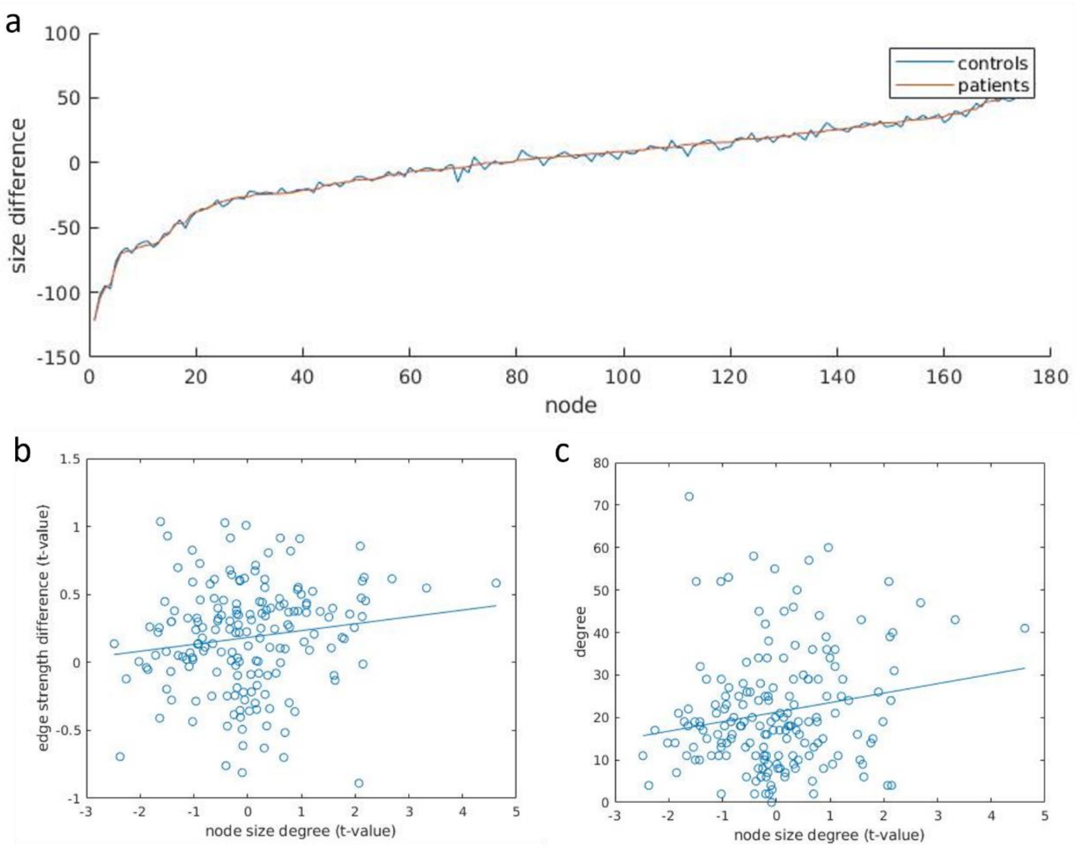

Figure 11 – Changes in parcels size and its correlation to node degree and edge dysconnectivity, based on s200 GSR. a The average difference in size of every region between individualized and group-based parcellation for patients and controls. Size is measured in terms of vertices and the change reported is the average size difference for controls and patients. Positive numbers correspond to the node being larger in individualized parcellation. There was no difference in parcel size changes between groups ($p = 0.981$). Panel **b** shows the node size difference and average edge dysconnectivity for every region (blue dots) and the correlation ($r = 0.160, p = 0.034$) between both (blue line) for individualized parcellation. Panel **c** shows the node size difference and node degree for every node (blue dots)

and the correlation ($r = 0.152, p = 0.043$) between both (blue line) for individualized parcellation. Correlation is given by the Spearman's coefficient.

Reference:

Xia, M., Wang, J., & He, Y. (2013). BrainNet Viewer: A Network Visualization Tool for Human Brain Connectomics. *PLOS ONE*, *8*(7), e68910. https://doi.org/10.1371/journal.pone.0068910
